# Commercial Cow Milk Contains Physically Stable Extracellular Vesicles Expressing Immunoregulatory TGF-β

**DOI:** 10.1371/journal.pone.0121123

**Published:** 2015-03-30

**Authors:** Bartijn C. H. Pieters, Onno J. Arntz, Miranda B. Bennink, Mathijs G. A. Broeren, Arjan P. M. van Caam, Marije I. Koenders, Peter L. E. M. van Lent, Wim B. van den Berg, Marieke de Vries, Peter M. van der Kraan, Fons A. J. van de Loo

**Affiliations:** Experimental Rheumatology, Radboud university medical center, Nijmegen, the Netherlands; Institut national de la santé et de la recherche médicale - Institut Cochin, FRANCE

## Abstract

**Scope:**

Extracellular vesicles, including exosomes, have been identified in all biological fluids and rediscovered as an important part of the intercellular communication. Breast milk also contains extracellular vesicles and the proposed biological function is to enhance the antimicrobial defense in newborns. It is, however, unknown whether extracellular vesicles are still present in commercial milk and, more importantly, whether they retained their bioactivity. Here, we characterize the extracellular vesicles present in semi-skimmed cow milk available for consumers and study their effect on T cells.

**Methods and Results:**

Extracellular vesicles from commercial milk were isolated and characterized. Milk-derived extracellular vesicles contained several immunomodulating miRNAs and membrane protein CD63, characteristics of exosomes. In contrast to RAW 267.4 derived extracellular vesicles the milk-derived extracellular vesicles were extremely stable under degrading conditions, including low pH, boiling and freezing. Milk-derived extracellular vesicles were easily taken up by murine macrophages *in vitro*. Furthermore, we found that they can facilitate T cell differentiation towards the pathogenic Th17 lineage. Using a (CAGA)12-luc reporter assay we showed that these extracellular vesicles carried bioactive TGF-β, and that anti-TGF-β antibodies blocked Th17 differentiation.

**Conclusion:**

Our findings show that commercial milk contains stable extracellular vesicles, including exosomes, and carry immunoregulatory cargo. These data suggest that the extracellular vesicles present in commercial cow milk remains intact in the gastrointestinal tract and exert an immunoregulatory effect.

## Introduction

Extracellular vesicles (EV), including exosomes, are membrane vesicles secreted by a variety of cells and are heterogeneous in size, ranging from 30–1000nm in diameter [1]. Although EVs were previously considered to be cellular waste products, EVs are now believed to be an important mediator in facilitating intercellular communication [2]. The production of EVs has been reported for many different cell types including macrophages, lymphocytes, dendritic cells, as well as epithelial and tumor cells [3–8]. EVs have been identified in a number of biological fluids such as blood, saliva and urine [9–14]. Recent studies also revealed that EVs are involved in the genetic exchange of RNA and microRNAs (miRNAs) between cells [15–17]. miRNAs are small (~22 nucleotide) non-coding RNAs that regulate gene expression at the post-transcriptional level [18]. In serum and saliva it is has been shown that the majority of miRNAs are present in microvesicles [19]. Exosome-like EVs have also been identified in both human breast milk and bovine colostrum [20,21]. It has been suggested that these breast milk-derived EVs are taken up systemically by the milk recipient, where they can play a role in the development of the infants immune system [22].

Infants are exposed to large numbers of foreign proteins, microorganisms and other chemicals and their resistance to infections relies heavily on the development of an appropriate immune system and on protective factors present in mother’s breast milk. Breast milk contains many components to promote development of neonatal immune competence, such as cytokines, antibodies and immune cells [23]. These immune-related factors not only provide passive immunity to infants, but can also modulate the development of the infant’s own immune system [24,25]. Among these cytokines is transforming growth factor beta (TGF- β), which is present in high amounts in a latent form [26,27]. Latent TGF- β in milk can be activated by gastric acid in the stomach [28]. Although the precise roles of TGF- β in milk remain poorly known, it has been suggested that TGF- β plays a role in the development of intestinal barrier function, initiation of IgA production and mucosal immunity during infancy [29,30]. It has also been shown that TGF- β in commercial milk provides protection against inflammation in mice [31].

In this study, we hypothesized that EVs containing immunoregulatory properties are present in commercial milk. To address this hypothesis, we isolated EVs from commercial milk and characterized their content. We found that milk-derived EVs carry both immune-related miRNAs and bioactive TGF- β. These milk-derived EVs were easily taken up by immune cells and were able to induce Th17 differentiation in murine splenocytes. To our knowledge, this is the first paper to show that commercial milk contains EVs carrying bioactive TGF- β which remains intact and active after exposure to low pH and can induce Th17 differentiation *in vitro*.

## Materials and Methods

### Milk samples and preparation of extracellular vesicles

Commercial semi-skimmed cow milk from a number of different brands was purchased at the local supermarket and stored at 4°C until EV isolation (within three days of purchase). Milk samples were centrifuged at 3.000g for 15 minutes at 4°C, to remove milk fat globules as well as any remaining mammary gland-derived cells. Defatted samples were then subjected to three successive centrifugations at 4°C for 1 h each at 12.000g, 35.000g, and finally at 70.000g to remove residual milk fat globules, casein proteins, and other debris. The supernatant was subsequently filtered through a 0.2μm syringe filter. EVs were isolated from the filtered supernatant, using ExoQuick reagent (System Biosciences, Bioconnect, Huissen, The Netherlands). For ExoQuick isolation, 63μl ExoQuick reagent was added to 250μl milk supernatant and precipitated overnight at 4°C. Precipitated samples were centrifuged at 1.500g for 30 minutes at 4°C, EV pellets were dissolved in either PBS or in the appropriate buffer for RNA or protein analysis. To isolate cellular EVs, macrophage (murine RAW 264.7-macrophages; ATCC TIB-71) culture medium was collected and centrifuged at 300g, subsequently supernatant was filtered through a 0.2μm syringe filter and precipitated with ExoQuick-TC (System Biosciences, Bioconnect, Huissen, The Netherlands). The amount of protein in each sample was measured with a Micro-BCA kit (Thermo Scientific, Pierce, Rockford, USA).

### Nanoparticle Tracking Analysis

Vesicle size distribution was estimated by the Brownian motion of the particles in a NanoSight LM12 using Nanoparticle Tracking Analysis 2.3 software (Nanosight Ltd, Amesbury, UK). Vesicles were diluted in PBS, till a suitable concentration for analysis was reached. Particle concentration was evaluated for the particles between 30–150nm in diameter.

### Electron microscopy

Milk-derived EVs were layered on top of a 30% sucrose cushion, and centrifuged for 70 minutes at 100.000g, to further purify the EVs [32]. After the sucrose cushion, the EV fraction was subjected to an additional ultracentrifugation step to pellet the EVs. EVs were taken up in small volumes of deionized water, which were placed on nickel grids and allowed to dry for 45 minutes. The grids with EVs were then washed by transferring them onto several drops of deionized water. Negative contrast staining was performed by incubating the grids on top of drops of 6% uranyl acetate. Excess fluid was removed and the grids were allowed to dry before examination on a Jeol JEM1400 Transmission Electron Microscope (Jeol, The Netherlands).

### Exosome capture assay

Antibodies against bovine-CD63 (1μg/ml) (MCA2042G, Bioconnect, Huissen, The Netherlands) were coated onto Maxisorp Nunc-Immuno plates (Thermo Scientific, Roskilde, Denmark) overnight at 4°C. Coated plates were blocked with 1% OVA 1h prior to addition of milk-derived EVs. Samples were diluted in blocking buffer and incubated overnight at 4°C. After incubation, the plates were washed multiple times with PBS-Tween. IgG1 was coated onto plates as a negative control for aspecific binding. After washing the plates 100μl of 100mM glycine-HCl (pH 2.4) was added to the wells to elute the bound EVs. After 30 minutes, the eluted samples were neutralized by the addition of NaOH. The diameters and concentration of the EVs were measured using a NanoSight LM12 (Nanosight Ltd., Amesbury, UK).

### Total RNA isolation

To confirm the presence of mRNA and miRNAs in milk vesicles, total RNA was isolated from the EV pellet using TRI reagent (Invitrogen, Carlsbad, CA) according to the manufacturer’s protocol. Briefly, 1.0ml TRI reagent and 200μl chloroform were added to the EV pellet and the mixture was vortexed for 15s and incubated at room temperature for 2 minutes. After centrifugation at 12.000g for 15 minutes at 4°C, the supernatant was transferred to a fresh tube and 500μl isopropanol was added. Ambion GlycoBlue Coprecipitant (Invitrogen, Carlsbad, CA) was added and samples were stored at -80°C overnight to improve the yield of total RNA, including miRNAs. After overnight incubation, the mixture was centrifuged at 12.000g for 30 minutes at 4°C to remove the supernatant and the RNA pellet was washed twice with 75% ethanol. Ethanol was removed by centrifugation at 12.000g for 5 minutes at 4°C. After pellets were air-dried for 10 minutes they were dissolved in 8μl RNase-free water.

### Quantitative real-time PCR

For the detection of RNA in EVs, the total RNA isolated was treated with DNaseI (Invitrogen, Carlsbad, CA). Synthesis of cDNA was accomplished by reverse transcription PCR an oligo(dT) primer and Moloney murine leukemia virus Reverse Transcriptase (Applied Biosystems Inc, Foster City, CA) and quantitative real-time PCR was performed using SYBR Green real-time PCR master mix on a Step-One according to the manufacturer’s instructions (Applied Biosystems Inc, Foster City, CA). Primer sets for individual genes, previously reported in bovine EVs [21], were used (see table 1). Standard curves were generated for each primer set to determine their efficiency. For miRNA detection, cDNA was generated from DNaseI treated RNA, using a QuantiMir RT Kit (System Biosciences, Mountain View, CA) according to the manufacturer’s instructions. PCR products were amplified using specific forward primers for miRNAs and the universal QuantiMir reverse primer, and detected using StepOne RT-PCR system (see table 2).

**Table 1:** Bovine RNA primer sequences.

Target	Accession No.	Sequence	Product (bp)
Beta-Casein	NM_181008	GGATTTCAAAGTGAATGCCCTGATGCAAGGATTGAAAAGTTG	80
Beta-Lactoglobulin	NM_173929	CGATGCCTTGAATGAGAACA TTTGTCGAATTTCTCCAGGG	163
Elongation Factor-1alpha	AB060107	ATTTGTGCCAATTTCTGGCT AGACATCCTGGAGAGGCAAA	194
GAPDH	BC102589	GGGTCATCATCTCTGCACCT ATCCACAGTCTTCTGGGTGG	215

**Table 2:** Bovine miRNA primer sequences.

Target	Accession No.	Sequence
bta-miR-21	MI0004742	AUGCUUAUCAGACUGAUGUUGACU
bta-miR-30a	MI0005054	UGUAAACAUCCUCGACUGGAAGC
bta-miR-92a	MI0009905	UAUUGCACUUCUGGGCCGGUCU
bta-miR-99a	MI0004751	AACCCGUAGAUCCGAUCUUGU
bta-miR-223	MI0009782	UGUCAGUUUGUCAAAUACCCCA

### Cellular vesicle uptake

Bovine milk-derived EVs were isolated as described above. Purified EVs were stained with PKH67 Green Fluorescent Cell Linker Kit for General Cell Membrane Labeling (Sigma-Aldrich, St. Louis, MO) as described previously [33], with minor modification in the washing process. In brief, EVs were diluted in PBS before addition of 300μl of Diluent C and 1μl PKH67. As a control, 300μl Diluent C and 1μl PKH67were added to PBS. The samples were mixed gently for 2 minutes before adding 500μl 1% BSA to bind excess dye. The samples were loaded onto 300 kDa Vivaspin filters (Sartorius Stedim Biotech GmbH, Goettingen, Germany) and centrifuged at 4.000g. The samples were washed three times with 2ml PBS, then taken up in culture medium. PKH67 labeled EVs (20μg/ml) were incubated with RAW macrophages for up to 24h at either 37°C, or 4°C as control, with 5% CO_2_. After incubation, cells were washed twice with PBS and fixed with 4% paraformaldehyde for 15 minutes at 4°C. The samples were washed twice with PBS and stained with 4,’6’-diaminido-2-phenylindole (DAPI; Vector Laboratories, Burlingame, CA). Cellular uptake of milk-derived EVs was observed using standard fluorescent and confocal microscopy (Leica microscopy, Rijswijk, The Netherlands). For confocal microscopy F4/80 (AbD Serotech) was used as a membrane staining, and DRAQ5 (eBioscience) for nuclei staining. For flow cytometric analysis macrophages, fibroblasts and primary adherent splenocytes were cultured for 24h at 37°C in the presence of PKH67 labeled EVs (20μg/ml). After culturing the cells they were washed twice with either PBS or citric acid buffer and analyzed by FACS.

### Luciferase reporter assay

Murine fibroblasts (NIH-3T3; ATCC CRL-1658) cells were seeded and rested for 24h prior to transduction with adenoviral CAGA_12_-luc construct (kindly provided by Peter ten Dijke, Dept. Molecular Cell Biology, Leiden University Medical Center, Leiden, The Netherlands). The CAGA_12_-boxes in the vector are transcribed by pSmad3/4 through active TGF-β receptor signaling, resulting in luciferase activity [34]. After another 24h, cells were starved for 6h in serum-free medium. After serum starvation, cells were stimulated for at least 20h with milk-derived EVs (20–200μg/ml), or recombinant human TGF- β 1 as control. Cells were lysed and luciferase activity was measured in lysates by Bright-Glo Reporter Assay System (Promega, Leiden, The Netherlands) in a luminescence microplate reader (BMG, Isogen life science, De Meern, The Netherlands). To validate the specificity for TGF- β, cells were incubated in the presence of 2,5μg/ml mouse anti-TGF- β 1,2,3 (MAB1835, R&D Systems, Abingdon, UK). Data is represented as relative luciferase units (RLU).

### Naïve T cell isolation and ex vivo T cell differentiation

Spleens were removed from 12–14 week old male DBA/1J mice (Janvier-Elevage, Saint Berthevin, France), mashed,passed through a 70μm strainer and erythrocytes were removed by osmotic shock. Cell suspension was re-suspended in 5% exosome-free fetal bovine serum containing RPMI1640 medium. To purify naïve splenic T cells, CD4+CD62L+ cells were isolated using MACS (Miltenyi Biotech, Leiden, The Netherlands). Purified naïve splenic T cells were cultured in the presence of plate-bound anti-CD3 (5μg/ml) and soluble anti-CD28 (2.5μg/ml) with anti-IL-2 (5μg/ml), IL-6 (50ng/ml), IL-1 β (10ng/ml), TNF-α (10ng/ml) (ITK diagnostics, Uithoorn, The Netherlands) and either EVs (400μg/ml) or TGF-β (1ng/ml) for four days. MAB1835 (5μg/ml) was used to determine TGF- dependence of Th17 differentiation. To assess Th17 differentiation, cells were re-stimulated for 4h with PMA (50 ng/ml) and Ionomycin (1μg/ml) (Sigma-Aldrich, St. Louis, MO), thereafter RNA was isolated to determine gene expression of IL-17 and ROR-γT. Primer used were as follows: GAPDH, Fw: 5’-GGC AAA TTC AAC GGC ACA-3’, Rv: 5’-GTT AGT TCA CTT CAA TTT GTG TCC TC-3’; IL-17A: Fw, 5’-CAG GAC GCG CAA ACA TGA-3’, Rv, 5’-GCA ACA GCA TCA GAG ACA CAG AT-3’; ROR-γT, Fw: 5’-CTG TCC TGG GCT ACC CTA CTG A- 3’, Rv: 5’-AAG GGA TCA CTT CAA TTT GTG TCC TC-3’.

### Ethical Statement

All animal tissue studies complied with national legislation and were approved by the local authorities of the Care and Use of Animals (DEC-nr: 2014–083).

### Statistical Analysis

All data are expressed as the mean ± SD or mean ± SEM. Data were compared using Mann-Whitney U test. Values of P < 0.05 were considered to indicate statistical significance. All statistical analyses were performed using GraphPad Prism 5.01 (GraphPad Software, La Jolla, CA).

## Results

### Characterization of milk-derived extracellular vesicles

**Fig 1:**
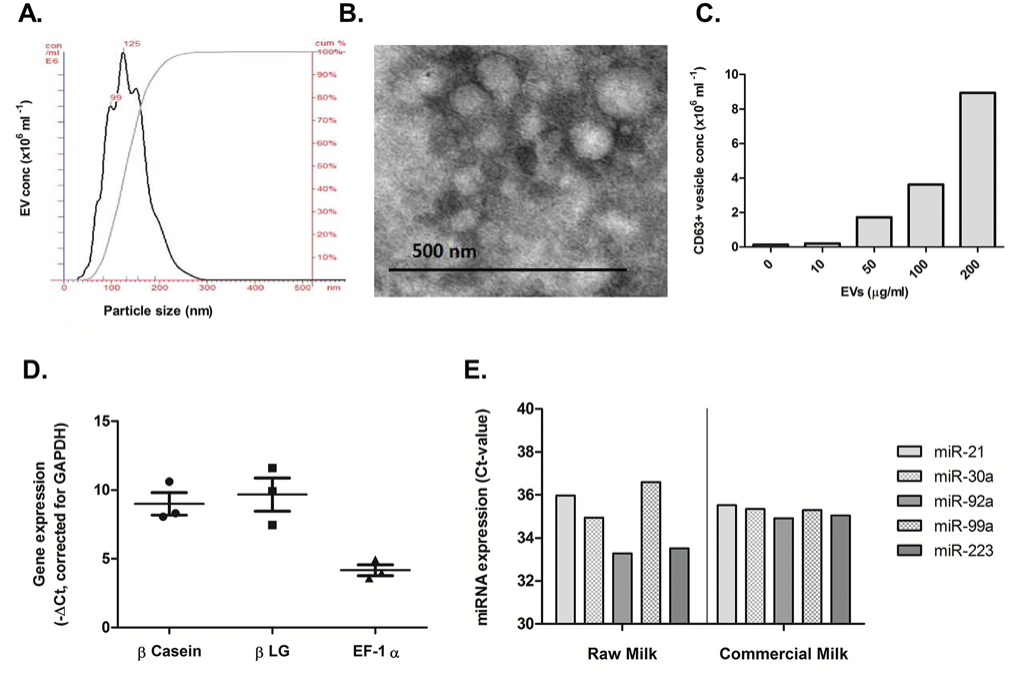
Characterization of bovine milk-derived extracellular vesicles. (A) Size distribution of isolated EVs observed in a NanoSight LM12. Raw data was analyzed with NTA software, with a minimum expected particle size of 50nm. At least 200 tracks had to be analyzed per sample for inclusion in final analysis. Data presented is a representation of >15 samples in multiple experiments. (B) Electron microscopy of the ultracentrifugation pellet from milk showed both exosomes with spherical shapes (30–100nm) and larger EVs ranging from 100–200nm. (C) Exosome capture assay, exosomes were captured with an anti-CD63 antibody, prior to elution and validation by NTA. (D) Detection of bovine specific RNA in EVs. β -Casein, β-Lactoglobulin (β LG) and elongation factor-1α (EF1α) were detected by RT-qPCR in both colostrum and commercial milk. (E) Detection of immunoregulatory miRNAs in EVs, including miR-21, miR-30a, miR-92a, miR-99a and miR-223.

To characterize milk-derived EVs from commercially available semi-skimmed milk, vesicles were isolated by differential centrifugation followed by ExoQuick preparation. Vesicles purified from milk were characterized using Nanoparticle tracking analysis (NTA) to determine size and particle concentration [35]. NTA showed that most EVs were between 100–150nm in diameter, with a peak at 125nm (Fig 1a). Particle concentration of milk EVs was roughly 5x10^7^ particles per μg protein. There were minimal differences between the different brands, regarding EV size and concentration. To confirm the presence of exosomes among our EVs, we looked at morphology and surface proteins of these vesicles. An examination of the purified EVs using electron microscopy revealed that they had the size (80–130nm) and morphology similar to that of exosomes (Fig 1b). To confirm the presence of exosomes among other milk-derived EVs, we used an exosome capture assay. Vesicles were captured on a coated high affinity plate with anti-CD63, a known exosome membrane marker, and milk-derived EVs were incubated overnight. After incubation, exosomes were eluted from the plate and measured with NTA (adapted protocol previously published by Jørgensen, *et al*. [36]). We found measureable levels of vesicles (Fig 1c), with an average size around 80nm. This confirmed the presence of exosomes in commercial milk among other larger EVs.

### Milk-derived extracellular vesicles contain both mRNA and miRNAs

We looked at the genetic content of the milk-derived EVs, by isolating total RNA containing both mRNAs and microRNAs. Real time PCR analysis was performed to examine the expression of several genes previously reported [21] to be present in bovine milk-derived EVs from colostrum and raw milk. We confirmed the presence of these mRNAs in milk-derived vesicles, as shown in Fig 1d (β -Casein, β -Lactoglobulin and elongation factor-1α). Subsequently, we analyzed miRNA content and found a number of immune-related miRNAs expressed in these vesicles, including miR-21, miR-30a, miR-92a, miR-99a and miR-223 (Fig 1e). We compared miRNAs between raw milk and commercial milk and found relatively small differences in expression levels. This indicates that a large portion of miRNAs remain present after processing.

### Milk-derived extracellular vesicles are very stable

**Fig 2:**
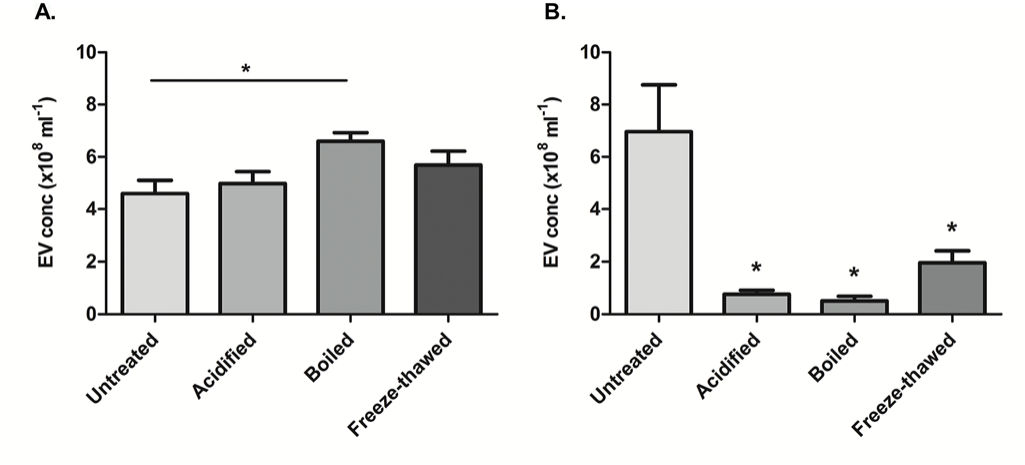
Milk-derived EVs are stable under degrading conditions. Extracellular vesicles (100μl, 200μg/ml) were acidified (pH = 2), boiled (15 minutes at 105°C) and frozen (liquid nitrogen) to determine their stability. Stability was assessed using Nanoparticle Tracking Analysis, by comparing particle size and concentration to untreated vesicles. (A) Milk-derived EVs showed no significant differences in concentration. (B) As a control, EVs isolated from macrophage culture supernatant were analyzed. The concentration of these vesicles was significantly reduced after all treatments. Statistically significant differences were determined by Mann-Whitney test, *p<0.*05*. Error bars represent mean ± S.D. (N = 4).

Previously, *Izumi et al*. [37] published that RNAs isolated from bovine colostrum are stable under degradative conditions. They hypothesized that these RNAs, carried by microvesicles, are stable enough to pass the intestinal tract of an infant after drinking breast milk. We examined the stability of our milk-derived EVs and compared these with EVs from cellular origin at low-pH (pH = 2.0), after boiling and multiple freeze-thawing cycles. We analyzed the EVs using NTA, to see if there were any changes in size distribution or particle concentration. The milk-derived EVs did not show any major differences, with comparable size (mode, mean and percentile undersize—data not shown) and concentration compared to untreated EVs (Fig 2a). However, in the EVs from cellular origin (Fig 2b) we found a significant reduction in particle concentration after acidification, boiling and freeze-thawing (90, 90 and 70%, respectively). This result strengthens the hypothesis that milk-derived EVs, carrying immunoregulatory cargo, are not degraded in the gastrointestinal tract.

### Cellular uptake by immune cells

**Fig 3:**
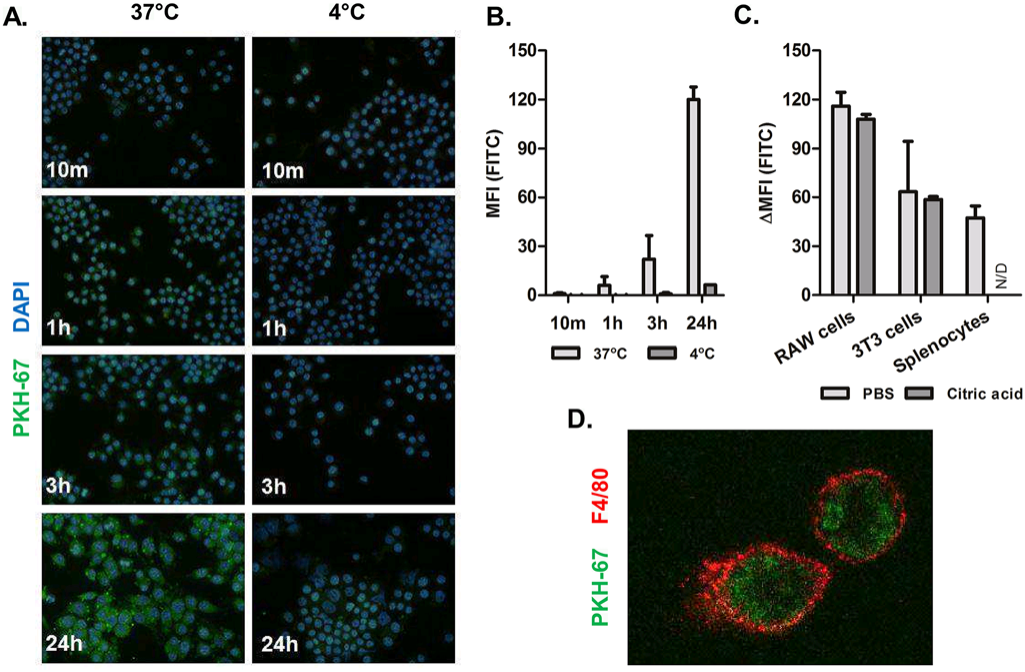
Cellular uptake of EVs in murine cells. Macrophages were incubated with PKH67-labeled milk-derived EVs (green) for various time points 37°C or 4°C as control for active uptake. (A) Images were obtained on a Leica fluorescent microscope and represent three separate experiments (magnification 400x). (B) Flow cytometric analysis for PKH67-staining (FITC wavelength) in macrophages was performed. The MFI of two separate experiments (performed in duplo) was averaged. (C) Flow cytometric analysis for PKH67-staining in RAW 264.7 macrophages and NIH 3T3 fibroblasts, washed with either PBS or citric acid to remove surface bound EVs and in primary adherent splenocytes. ΔMFI was corrected for unstained EVs in culture. (D) Confocal microscopy confirmed intracellular uptake of EVs, membranes were stained with F4/80 (red) (magnification 2000x). N/D means not done. Error bars represent mean ± S.D. (N = 3).

Uptake of milk-derived EVs by phagocytic macrophages (RAW 264.7) was assessed using EVs labeled with a fluorescent dye, PKH67, which was previously published to be a suitable labeling technique for exosomes and EVs [33]. PKH67 labeled EVs were incubated with macrophages for 10m, 1h, 3h and 24h and cellular uptake was examined using fluorescent microscopy and flow cytometric analysis (Figs 3a and 3b, respectively). As a control for active uptake, cells were incubated at 4°C and uptake was assessed. Both fluorescent microscopy and flow cytometry showed uptake of milk-derived EVs in macrophages, to a similar extent. In both cases the 4°C control did not show significant uptake, excluding passive uptake or sticking of vesicles. Cellular uptake was also assessed for fibroblast cells (NIH 3T3) and primary murine splenic adherent cells (Fig 3c). Milk-derived EVs were taken up by all cell types, but to different extents. To confirm that these vesicles were taken up by the cells and not only bound to the cell membrane, cells were washed with citric acid, which has previously been published as a suitable method to wash away surface-bound EVs [33]. As shown in Fig 3c, washing the cells with citric acid did not change the fluorescent intensity, confirming that the EVs were taken up. We also performed confocal laser microscopy, to show that EVs are inside the cells. A representation is shown in Fig 3d, where it is clearly visible that the vesicles are inside the cytoplasm of the macrophages. Taken together, these data suggest that milk-derived EVs are easily taken up by different cell types.

### Active TGF-β present on milk-derived extracellular vesicles

**Fig 4:**
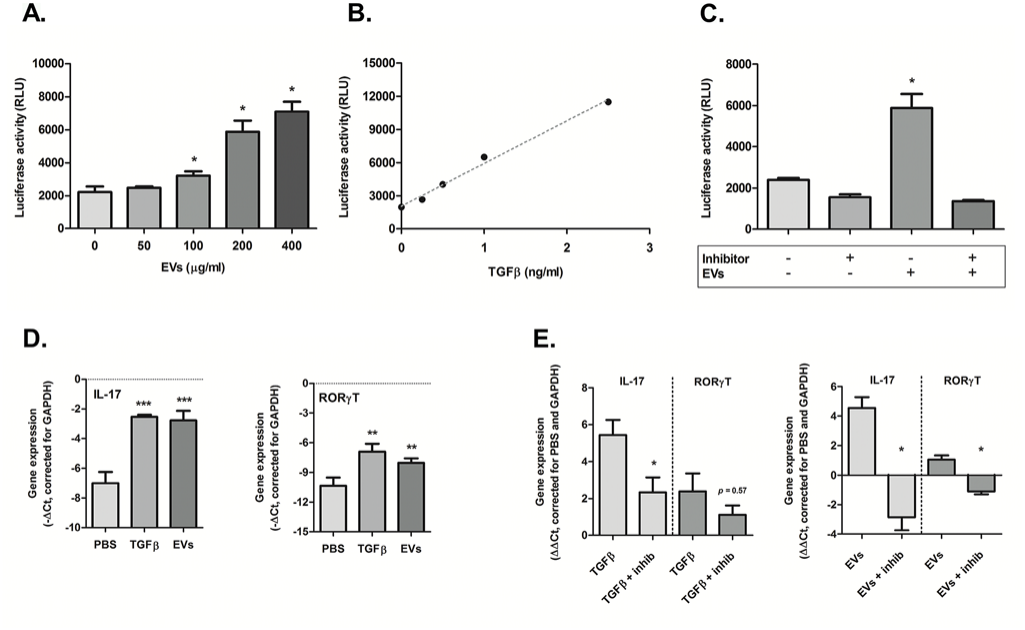
Active TGF-β is present on milk-derived extracellular vesicles. Milk EVs were added to NIH 3T3-cells transduced with CAGA_12_-luc construct. After 20h of culture, the reporter cells were lysed and the luciferase activity was measured. (A) A dose response of pSmad3/4-signaling was induced by the incubation with milk EVs. (B) Standard curve using recombinant rhTGF-β 1. (C) Blocking active TGF-β using an anti-TGF-β 1,2,3 antibody (2,5 μg/ml) abolished the induction of Smad-signaling by milk EVs. (D) Induction of Th17 differentiation, facilitated by TGF-β (1ng/ml) as positive control or milk EVs (400μg/ml), measured by the increased expression of ROR-γT and IL-17 mRNA compared to stimulation in absence of both N = 4 mice). (E) Blocking active TGF-β using an anti-TGF-β1,2,3 antibody (5μg/ml) inhibits Th17 differentiation (N = 4 groups, 3 mice in each group). Statistically significant differences were determined by Mann-Whitney test, *p<0.*05*, **p<0.*01*, ***p<0.*005*. Error bars represent mean ± S.D.

Expression of TGF-β on the surface of tumor-derived exosomes has been published previously [38]. Latent TGF-β in milk can be activated by gastric acid in the stomach [28]. It has also been shown that TGF-β in commercial milk provides protection against inflammation in mice [31]. However, it is unknown if TGF-β is also associated with EVs isolated from milk. Therefore we isolated milk-derived EVs and used the CAGA_12_-luc assay, a luciferase reporter construct that is activated by transcription factors down-stream of TGF-β receptor signaling, to determine levels of active TGF- β on the vesicles. We confirmed the presence of active TGF-β in a dose-dependent response with increasing amounts of milk-derived EVs added to the reporter cells (Fig 4a). A standard curve was made using recombinant human TGF-β 1 (Fig 4b). Milk-derived EVs contained roughly 3–3.5pg active TGF-β per 1μg EV protein. To test whether the effects were TGF-β specific, we blocked active TGF-β using MAB1835. Milk-derived EVs in combination with the antibody did no longer activate the CAGA_12_-luc reporter cells (Fig 4c), confirming that the effects were indeed TGF- β mediated. These results show that there are measurable levels of active TGF- β present on milk-derived EVs, which can induce Smad-signaling upon binding to the TGF-β receptor.

### Th17 differentiation induced by milk-derived extracellular vesicles

After finding active TGF-β on commercial milk-derived EVs, we hypothesized that they could modulate T cell differentiation and thereby play a role in the immune system. It is known that TGF-β is required for the induction of the pathogenic T-helper17 cells (Th17) [39,40] under inflammatory conditions. To test this hypothesis we isolated naïve T cells from murine spleens and incubated them with a Th17 differentiation cocktail without TGF-β and instead added milk-derived EVs. After four days in culture mRNA was isolated and Th17 differentiation was analyzed using RT-qPCR. Significant increases of gene expression for both ROR-γT and IL-17 (*p<0*.*01* and *p<0*.*005*, respectively) were observed in the presence of milk-derived EVs compared to cells stimulated in the absence of TGF-β (Fig 4d). To confirm the Th17 differentiation was dependent on TGF-β present on the EVs, we used MAB1835. Blocking TGF-β significantly inhibited (*p<0*.*02*) Th17 differentiation induced by milk EVs (Fig 4e).

## Discussion

In the present study, we confirmed the presence of a large amount of EVs in commercial milk, we estimate that semi-skimmed milk contains 5x10^10^ vesicles per ml. EVs, including exosomes, are known to be present in human and bovine colostrum and mature milk [20,21]. Extracellular vesicles isolated from commercial milk have a similar RNA and miRNA content compared to microvesicles isolated from bovine colostrum [21]. The presence of EVs in commercial milk indicates that they are very stable and can withstand the harsh conditions during pasteurization. It is known from literature that the lipid bilayer of milk exosomes can protect their miRNA-cargo against harsh degrading conditions like low acidic pH and RNase-mediated degradation [25,37]. We found that milk-derived EVs were insusceptible to degradation during treatment at low pH, boiling and freezing, unlike macrophage-derived EVs, thereby confirming the stability of milk-derived EVs. In nucleated fish erythrocytes the importance of extracellular calcium for membrane stability and integrity has been shown[41]. A general idea is that Ca^2+^, by binding to phospholipids, stabilizes lipid bilayers and thus provides structural integrity to cellular membranes. The high calcium levels found in milk might therefore contribute to the higher stability of milk-derived EVs, however further research is required to confirm whether this holds true for EVs. The high stability of the milk-derived EVs at low pH suggests they are stable enough to pass through the gastrointestinal tract of an infant. This hypothesis is in line with a recent review by Melnik, *et al*. [22] on milk as a possible genetic transfection system, they mentioned that milk exosomes most likely reach systemic circulation. In fact, it has already been demonstrated that plant-derived miR-168a, found in the diet of humans, reaches the plasma of human subjects and can affect LDLRAP1 metabolism in the liver [42]. Although these findings have been met with skepticism from other researchers in the field, and therefore extrapolation of the results should be done with caution [43,44].

The presence of immunoregulatory miRNAs suggests that milk-derived EVs can influence the milk recipient’s immune system. Several immunoregulatory miRNAs have already been reported to be expressed in mammary gland cells and in EVs isolated from both bovine and human milk [21,45]. If miRNA, present in raw milk EVs, influence the milk recipient’s immune system this will most likely also be the case for commercial milk EVs. When comparing the expression pattern of miRNAs in EVs isolated from commercial and raw milk we found only minor differences in expression levels. In addition to immunoregulatory miRNAs, we also found active TGF-β on these vesicles. Although the precise roles of TGF-β in milk remain poorly known, there is a relation between milk consumption and reduced incidence of atopic diseases due to increased levels of regulatory T cells in infants [46,47]. The expression of TGF-β on milk-derived EVs might contribute to the higher levels of regulatory T cells in these infants, as TGF-β is known to be an important regulating cytokine during differentiation into regulatory T cells [48,49]. A recent study by Cai, *et al*. [50] has in fact shown that exosomes from TGF-β1 gene-modified dendritic cells are immunosuppressive and can induce regulatory T cells. In a human study [20], it has already been shown that exosomes isolated from breast milk can induce regulatory T cells in PBMCs, however, the mechanism was not mentioned. Besides the role in regulatory T cells, TGF-β is also important in the differentiation of T cells towards Th17 cells [39,40]. Under inflammatory conditions, TGF-β can induce Th17 cells [51]. We also found that milk-derived EVs are able to induce significant Th17 differentiation when incubated in combination with a pro-inflammatory cytokine cocktail. The proinflammatory activity of Th17 cells can be beneficial to the host during infection [52–54]. However, inappropriate or uncontrolled Th17 activation has been linked to several autoimmune and inflammatory pathologies [55–58]. The number of EVs (and thereby amount of TGF-β) we chose to introduce into our T cell culture is most likely too low to induce significant Treg differentiation, however, addition of more EVs in combination with a different cytokine cocktail might potentiate Treg differentiation in an *in vitro* setting. Therefore, we hypothesize that milk-derived EVs could play different roles in health and disease. The induction of regulatory T cells can be beneficial to infants and healthy individuals, however, during high levels of inflammation seen in many autoimmune diseases milk-derived EVs could promote pathogenic Th17 cell differentiation.

In conclusion, the results of the present study demonstrates that milk-derived EVs, including exosomes, are present in commercial cow milk. These vesicles are extremely stable, and can facilitate the differentiation of naïve T cells into pathogenic Th17 cells due to carrying bioactive TGF-β. This is an important finding as consuming commercial milk might expose humans to immunoregulatory EVs and this could have implications in health and disease.
